# AGE DIFFERENCES IN EMOTION-RELATED OUTCOMES OF COGNITIVE ENGAGEMENT AND MEANINGFULNESS AT WORK

**DOI:** 10.1093/geroni/igad104.3119

**Published:** 2023-12-21

**Authors:** Melanie Johnson, Claire Growney, Li Chu, Laura Carstensen

**Affiliations:** San Diego State University, San Diego, California, United States; Stanford University, Stanford, California, United States; Stanford University, Stanford, California, United States; Stanford University, Stanford, California, United States

## Abstract

Research suggests that there are few age differences in the enjoyment of cognitive engagement, but the importance of engagement in emotionally meaningful goals increases with age. In the present study we examined the interplay between cognitive engagement, meaningfulness at work, and related emotional states in older and younger workers. We used a five-day modified day reconstruction method in which 203 employees (aged 25–76) described their work, emotions, and cognitive engagement during the beginning, middle, and end of their workdays. A three-way interaction revealed that older employees who were more cognitively engaged at work reported higher levels of negative emotions than younger employees, and the association was reduced among those who perceived their work as meaningful (b=–0.006, SE=0.003, p<.05). Interestingly, perceived meaningfulness was a stronger predictor of pride at work among younger employees than older employees (b = –0.015, SE = 0.006, p = .018). Cognitive engagement at work was predictive of feeling pride among older employees, and not younger employees (b=0.02, SE=0.01, p<.01). Age also moderated the relationship between cognitive engagement and time-savoring, such that higher levels of cognitive engagement predicted more time-savoring at work among older employees, but not younger employees (b=0.011, SE=0.005, p< 05). Results suggest that the emotional benefits of perceiving one’s work as meaningful may be different for younger and older workers. Furthermore, associations between cognitively engaging work and emotional outcomes such as time-savoring and pride may be especially relevant for older employees.

